# Catalytic mechanism of the tyrosinase reaction toward the Tyr^98^ residue in the caddie protein

**DOI:** 10.1371/journal.pbio.3000077

**Published:** 2018-12-31

**Authors:** Yasuyuki Matoba, Shogo Kihara, Naohiko Bando, Hironari Yoshitsu, Miyuki Sakaguchi, Kure’e Kayama, Sachiko Yanagisawa, Takashi Ogura, Masanori Sugiyama

**Affiliations:** 1 Graduate School of Biomedical & Health Sciences, Hiroshima University, Hiroshima, Japan; 2 Picobiology Institute, Graduate School of Life Science, University of Hyogo, Hyogo, Japan; Georgia Institute of Technology, UNITED STATES

## Abstract

Tyrosinase (EC 1.14.18.1), a copper-containing monooxygenase, catalyzes the conversion of phenol to the corresponding ortho-quinone. The *Streptomyces* tyrosinase is generated as a complex with a “caddie” protein that facilitates the transport of two copper ions into the active center. In our previous study, the Tyr^98^ residue in the caddie protein, which is accommodated in the pocket of active center of tyrosinase, has been found to be converted to a reactive quinone through the formations of the μ-η^2^:η^2^-peroxo-dicopper(II) and Cu(II)-dopasemiquinone intermediates. Until now—despite extensive studies for the tyrosinase reaction based on the crystallographic analysis, low-molecular-weight models, and computer simulations—the catalytic mechanism has been unable to be made clear at an atomic level. To make the catalytic mechanism of tyrosinase clear, in the present study, the cryo-trapped crystal structures were determined at very high resolutions (1.16–1.70 Å). The structures suggest the existence of an important step for the tyrosinase reaction that has not yet been found: that is, the hydroxylation reaction is triggered by the movement of Cu^A^, which induces the syn-to-anti rearrangement of the copper ligands after the formation of μ-η^2^:η^2^-peroxo-dicopper(II) core. By the rearrangement, the hydroxyl group of the substrate is placed in an equatorial position, allowing the electrophilic attack to the aromatic ring by the Cu_2_O_2_ oxidant.

## Abstract 2

The cryo-trapped crystal structures of tyrosinase in a complex with its “caddie” protein reveal structural insight into the catalytic mechanism of tyrosinase, the rate-limiting enzyme in the production of melanin.

## Author summary

Tyrosinase is an enzyme that controls a rate-limiting reaction of melanogenesis: it catalyzes the conversion of a phenol to the corresponding ortho-quinone. *Streptomyces* tyrosinase is formed as a complex, with a “caddie” protein that assists with the transport of the two copper ions into the enzyme’s active center. In our previous study, we showed that the Tyr^98^ residue in the caddie protein, which is accommodated in the pocket of active center of tyrosinase, is converted to a reactive quinone through the formations of the μ-η^2^:η^2^-peroxo-dicopper(II) and Cu(II)-dopasemiquinone intermediates. Until now—despite extensive studies of the tyrosinase reaction based on the crystallographic analysis, low-molecular-weight model systems, and computer simulations—the catalytic mechanism was unclear at an atomic level. To understand the catalytic mechanism of tyrosinase in detail, we determined the cryo-trapped crystal structures at very high resolutions, which suggest an important new step for the tyrosinase reaction: the hydroxylation reaction triggered by the movement of Cu^A^, which induces the syn-to-anti rearrangement of the copper ligands after the formation of μ-η^2^:η^2^-peroxo-dicopper(II) core.

## Introduction

**Fig 1:**
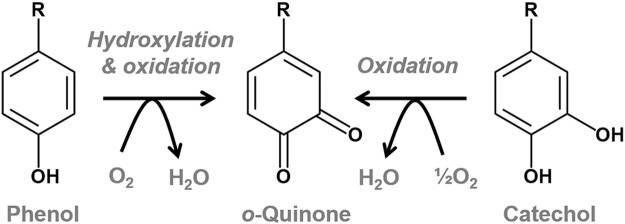
Reactions catalyzed by tyrosinase.

Tyrosinase (EC 1.14.18.1), which has an active center formed by dinuclear copper, catalyzes the conversion of phenol to the corresponding ortho-quinone through the hydroxylation and subsequent oxidation reactions, together with the oxidation of catechol to the quinone [1–6] (Fig 1). The quinone product is a reactive precursor to synthesize melanin. A series of reactions is coupled with reduction of dioxygen to water.

Tyrosinase is a type 3 copper protein family including catechol oxidase [7] and hemocyanin [8]. Although the former enzyme oxidizes catechol to the corresponding quinone, it lacks a monooxygenase activity. On the other hand, hemocyanin acts as a dioxygen carrier in arthropods and mollusks. The dicopper center of the type 3 copper protein takes three redox forms [1–6]. The deoxy form [Cu(I)–Cu(I)] has two cuprous ions into the active center, which binds dioxygen to yield the oxy form. In the oxy form, dioxygen binds as a peroxide ion in a μ-η^2^:η^2^ side-on bridging mode [Cu(II)–O_2_^2-^–Cu(II)]. The met form [Cu(II)–Cu(II)] denotes a state in which copper atoms at the active site are oxidized, but dioxygen is not bound to the copper atoms. As for tyrosinase, the met form is a resting enzymatic form, in which two cupric ions are bridged with one or two small ligands, such as water molecules or hydroxide ions. The oxy form catalyzes the conversion of the phenol and catechol substrates to ortho-quinones, whereas the met form does not catalyze the former reaction containing an oxygenation step [1].

Our group has previously cloned a melanin-synthesizing gene cluster from the *Streptomyces* (*S*.) *castaneoglobisporus* HUT6202, which produces a large amount of melanin [9]. The cluster is composed of two cistrons: one is an open reading frame consisting of 378 nucleotides and designated as *orf378*. The other gene designated *tyrC*, which is located just downstream of *orf378*, encodes tyrosinase. Because the *orf378* gene product facilitates the incorporation of copper ions to the apo-tyrosinase, we named it as a “caddie” protein. As observed in the case of the *S*. *antibioticus* tyrosinase and its partner protein, MelC1 [10], the Cu(II)-free tyrosinase forms a complex with the caddie protein [11]. Although the tyrosinase is not activated by copper added from the outside, the addition of copper to the complex facilitates the incorporation of two copper ions into tyrosinase. Furthermore, the resulting Cu(II)-bound tyrosinase is liberated from the complex, whereas the released caddie protein is not detectable in a solubilized fraction, suggesting that the released caddie molecules form aggregation.

**Fig 2:**
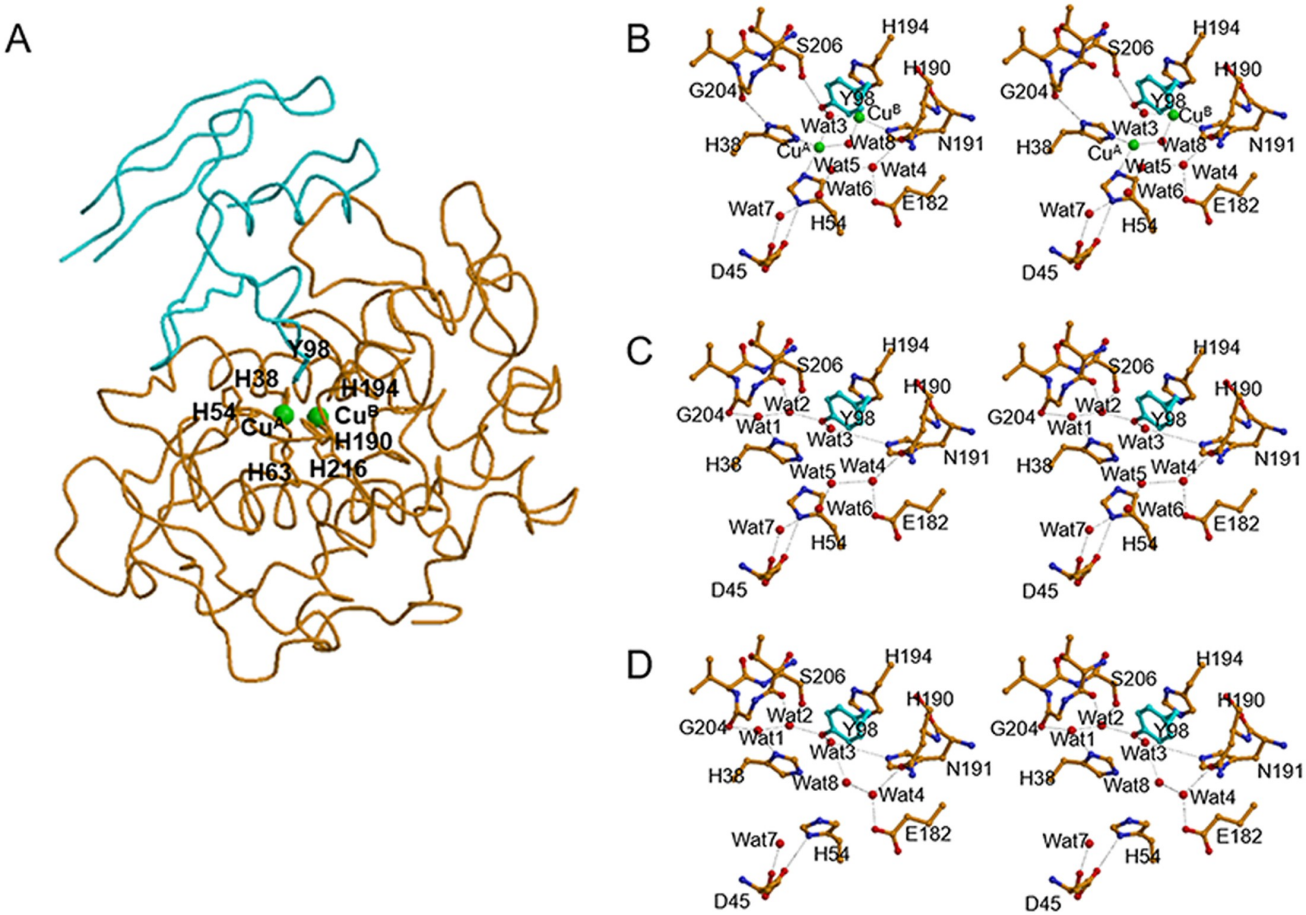
The structure of tyrosinase in a complex with the caddie protein. (A) Overall structure. Tyrosinase and the caddie protein are shown in orange and cyan, respectively. Copper ions (Cu^A^ and Cu^B^) identified at the catalytic site are indicated by green spheres. Residues of the ligand for the copper ions and the Tyr^98^ residue in caddie are shown as a stick model. The His^54^ residue of tyrosinase, which is a ligand for Cu^A^, takes two conformations even in the met2 form. (B) A structural model around the catalytic site of the met2 form of Cu(II)-bound tyrosinase. One of the two conformations of His^54^, which is unbound to Cu^A^, is omitted from the model for the convenience. (C and D) Structural models of the catalytic site of Cu(II)-free tyrosinase, in which the side chain of the His^54^ residue takes conformations orienting toward the Cu^A^-binding site and the surface of the caddie protein, respectively. In (B) to (D), carbon atoms from the residues of tyrosinase and the caddie are represented by orange and cyan, respectively. Copper ions and water molecules (“Wat”) are represented by green and red spheres, respectively. His^63^ and His^216^ residues are omitted from the model, for convenience. E, glutamic acid; D, aspartic acid; G, glycine; H, histidine; N, asparagine; S, serine; Y, tyrosine.

We have determined the tertiary structure of the *S*. *castaneoglobisporus* tyrosinase in a complex with the caddie protein at very high resolutions [12] (Fig 2). This is the first determination of the crystal structure of tyrosinase, demonstrating its structural similarity with the catechol oxidase previously determined (Protein Data Bank [PDB] ID: 1BT1) [13]. The crystal structure of the Cu(II)-free tyrosinase in the complex with the caddie (PDB ID: 1WXC) was determined at 1.20-Å resolution. We have obtained the met forms of Cu(II)-bound tyrosinase complexed with the caddie protein by soaking the native crystals in a CuSO_4_-containing solution. At the active center of tyrosinase, each of two closely positioned copper ions (Cu^A^ and Cu^B^) is surrounded by three histidine residues through the Nε nitrogen atoms. The met1 form (PDB ID: 1WX3 at 1.33-Å resolution), which was obtained by soaking for about 40 h, has a molecule containing one oxygen atom between the copper ions, whereas the met2 form (PDB ID: 2AHK at 1.71-Å resolution), which was obtained by soaking for longer than 80 h, has two molecules. In the crystal structure of the complex [12], the Tyr^98^ residue of the caddie protein is present in the active-site pocket of tyrosinase (Fig 2). In recent years, several crystal structures of tyrosinase-related enzymes have been elucidated, e.g., prophenoloxidases from the insect *Manduca sexta* (PDB ID: 3HHS) [14] and *Anopheles gambiae* (PDB ID: 4YZW) [15], tyrosinases from the bacterium *Bacillus megaterium* (PDB ID: 3NM8) [16] and the fungus *Agaricus bisporus* (PDB IDs: 2Y9X and 4OUA) [17,18], and protyrosinase (MelB, PDB ID: 3W6Q) [19] and small catechol oxidase (PDB ID: 4J3P) [20] from the fungus *Aspergillus oryzae*. Additionally, the crustacean and plant enzymes with tyrosinase-like activity have been crystallographically characterized (PDB IDs: 2P3X, 3WKY, 4Z0Y, 5CE9) [21–24], as has the X-ray structure of human tyrosinase-related protein 1 (TRP1) (PDB ID: 5M8T) [25].

At the dicopper center of tyrosinase from *S*. *castaneoglobisporus*, Cu^A^ is surrounded by His^38^, His^54^, and His^63^ residues, whereas Cu^B^ is surrounded by His^190^, His^194^, and His^216^ residues [12] (Fig 2A and 2B). In the absence of copper ions, the His^54^ residue takes two conformations that are suggested to be important for the copper acquisition [26]. When the side chain of His^54^ is oriented toward the Cu^A^-binding site, seven water molecules may be present in the active center (Fig 2C). Four of the water molecules (Wat^4^–Wat^7^) are aligned between the side chain of Asn^191^ and the main-chain carbonyl of Asp^45^. The Wat^4^ molecule forms hydrogen bonds with the side-chain atoms of Glu^182^ and Asn^191^. The Glu^182^ residue is well conserved among tyrosinase enzymes, whereas the Asn^191^ is less conserved. A recent example is provided by two tyrosinases from *Malus domestica* that present an alanine or a glycine at the position corresponding to Asn^191^ [27]. However, it was reported that the replacement of the residue corresponding to Asn^191^ to glycine significantly reduced the tyrosinase activity [28]. The side chain of the caddie Tyr^98^ residue forms hydrogen bonds with Wat^2^ and Wat^3^. Wat^1^ exists between the side chain of His^38^ and the main-chain carbonyl of Gly^204^. On the other hand, when the side chain of His^54^ protrudes toward that of the surface residue in the caddie protein, six water molecules are present in the active center (Fig 2D). In detail, two waters (Wat^5^ and Wat^6^) are removed to avoid the close contact with the side chain of His^54^. Instead, Wat^8^ is introduced between Wat^3^ and Wat^4^. In the met2 form, two copper ions are present at the Cu^A-2^- and Cu^B-2^-binding sites at a distance of 3.4 Å, with two bridging molecules. The bridging molecules (presumably two hydroxide ions) are positioned at the Wat^3^ and Wat^8^ sites. The His^54^ residue is disordered even in the met2 form, probably because of the steric hindrance between His^54^ and Wat^8^ [26]. In addition, the Wat^1^ and Wat^2^ molecules were found to disappear from the active center, and the side chains of His^38^ and caddie Tyr^98^ were altered to interact directly with Gly^204^ and Ser^206^, respectively. Although the functional meaning of the disappearance of water molecules, which is coupled with the introduction of copper ions, is currently unclear, it may be an advantage in the entropic energy term.

In the previous study, we have discussed the transferring mechanism of Cu(II) ions to the active center of tyrosinase, which is assisted by the caddie protein, on the basis of the kinetic and crystallographic studies [26]. The binding sites for the additional copper ions (Cu^C^, Cu^D^, and Cu^E^) in the caddie protein and the hydrogen-bonding network around the tyrosinase active site were found to be important for the effective transfer of Cu(II). Our group has recently demonstrated that the incorporation of copper ions into tyrosinase and the following release of copper-bound tyrosinase progress more quickly in the presence of NH_2_OH, which can reduce the met form to the deoxy form, but not the oxy form, under aerobic conditions [29]. Cu(I), but not Cu(II), must be suitable species to be incorporated into the active center of tyrosinase. Furthermore, the mass spectroscopic analysis has indicated that the Tyr^98^ residue in the caddie protein is converted to the reactive dopaquinone, which stimulates the aggregation of the caddie protein and the dissociation of tyrosinase from the complex. The dopaquinone must be formed as a result of the catalytic activity of the oxy-tyrosinase. The ultraviolet-visible (UV-vis) and resonance Raman spectroscopic analyses indicated that the Tyr^98^ residue is converted to dopaquinone through the formations of μ-η^2^:η^2^-peroxo-dicopper(II) and Cu(II)-dopasemiquinone intermediates [29], although the formation of dopaquinone is a speculation from the fact that the modified caddie is easily aggregated. Reaction intermediates were able to be trapped under the conditions at which the aggregation of the caddie was inhibited.

Until now—despite extensive studies based on the crystal structures of tyrosinase [12,15,23,24,28,30–33], low-molecular-weight model systems [4–6,34–39], or computer simulations [40–42]—its catalytic mechanism has not yet been clearly understood at an atomic level. For instance, we need to understand about the oxidation states of copper ions, the bases in the tyrosinase reaction, and the lack of tyrosine hydroxylase activity in catechol oxidases. To understand the catalytic mechanism, in the present study, we analyzed time-resolved X-ray crystal structures of the complex between tyrosinase and caddie after the addition of a reducing agent under aerobic conditions.

## Results

**Fig 3:**
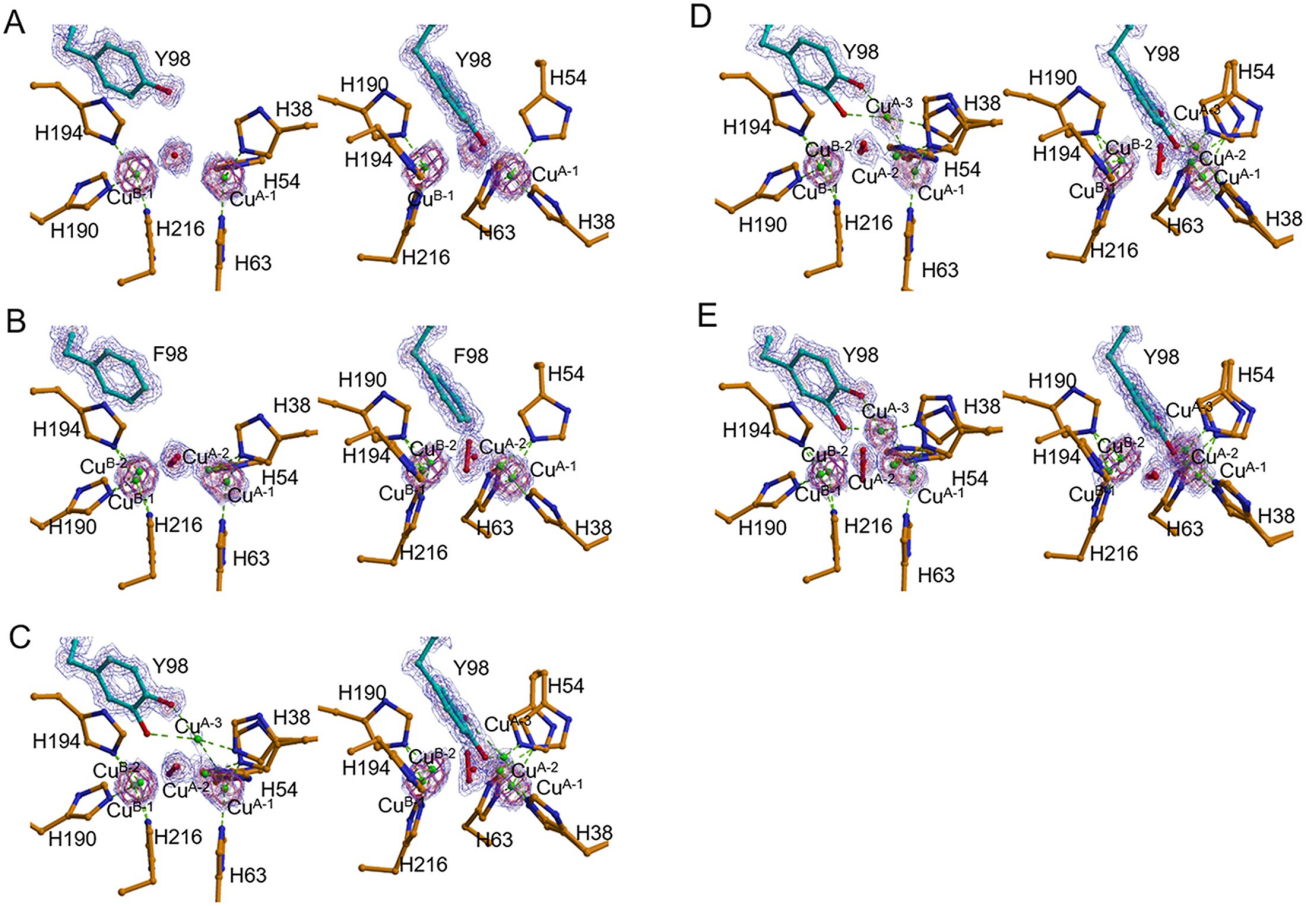
Electron density around the dicopper center and 98th residue in the caddie protein obtained by the NH_2_OH treatment to the met2-form crystals. (A) was obtained by the anaerobic soaking of the wild-type complex with CuSO_4_ and NH_2_OH for 2 h (ST1). (B) was obtained by the aerobic soaking of the Y98F-mutated complex for 2 h (ST2). (C), (D), and (E) were obtained by the aerobic soaking of the wild-type complex for 10 min (ST3), 20 min (ST4), and 2 h (ST5), respectively. The 2*F*_o_-*F*_c_ electron density map around Tyr^98^ (or Phe^98^) in the caddie protein, Cu^A^, Cu^B^, and bridging molecules was contoured at 1.5 σ (blue), 2.5 σ (purple), 3.5 σ (orchid), 4.5 σ (violet red), and 5.5 σ (red). The right panels are different views of the left panels. Electron densities for a hydroxyl group newly added to the Tyr^98^ residue in ST3 or ST4 are invisible in the present maps because of the low occupancy. H, histidine; F, phenylalanine; Y, tyrosine; Y98F-mutated complex, complex between tyrosinase and the mutated caddie, in which the Tyr^98^ residue is replaced with phenylalanine.

In the present study, the deoxy-tyrosinase complexed with the caddie protein (ST1) was obtained by soaking the met2-form crystal in a purged solution containing NH_2_OH for 2 h anaerobically at 25 °C (Table 1). The use of synchrotron radiation improved the resolution, when compared with the deoxy form reported previously (PDB ID: 2AHL at 1.60-Å resolution) [12]. Similar to the results obtained previously, two copper ions are at the Cu^A-1^- and Cu^B-1^-binding sites at a distance of 4.3 Å in ST1 (Fig 3A). The bridging molecule, which may be a water molecule, is positioned at the Wat^3^ site. Both Cu^A-1^ and Cu^B-1^ take a trigonal coordination with three Nε atoms from the histidine residues in each, rather than a tetrahedral one, since the bridging molecule is somewhat distant from both Cu^A-1^ and Cu^B-1^ (S1A Fig).

**Table 1:** Data collection and refinement statistics.

Data set	ST1	ST2	ST3	ST4	ST5	ST6
Protein	WT	Y98F	WT	WT	WT	H63F
Preparation^a^	anaerobic	aerobic	aerobic	aerobic	aerobic	aerobic
	25 °C, 2 h	25 °C, 2 h	25 °C, 10 min	25 °C, 20 min	25 °C, 2 h	25 °C, 24 h
Data collection						
Beam line	BL26B2	BL26B2	BL26B2	BL38B1	BL26B2	BL26B2
Wavelength (Å)	0.90000	0.90000	0.90000	1.00000	0.90000	0.90000
Space group	*P*2_1_2_1_2	*P*2_1_2_1_2	*P*2_1_2_1_2	*P*2_1_2_1_2	*P*2_1_2_1_2	*P*2_1_2_1_2
Cell dimensions (Å)						
*a*	64.66	64.63	64.65	65.20	64.77	64.91
*b*	97.01	96.93	96.99	97.79	96.98	97.35
*c*	54.83	54.51	54.87	55.10	54.55	54.92
Resolution (Å)	50–1.16	50–1.16	50–1.16	100–1.32	100–1.18	100–1.70
Unique reflection	118,873	117,886	118,976	83,312	113,464	35,544
Redundancy^b^	7.0 (5.4)	6.7 (4.9)	6.9 (5.7)	6.9 (6.9)	7.1 (5.8)	6.0 (5.1)
Completeness (%)^b^	99.4 (96.1)	99.1 (94.5)	99.2 (97.8)	99.9 (100)	99.9 (99.9)	90.7 (91.8)
*R*_merge_ (%)^b^^,^^c^	4.8 (39.2)	5.0 (41.2)	4.2 (37.2)	6.5 (42.6)	5.8 (42.3)	5.8 (44.2)
*I*/σ^b^	38.2 (2.9)	37.0 (2.5)	41.8 (3.4)	16.6 (4.8)	34.2 (2.9)	28.8 (2.7)
Refinement						
Resolution (Å)	30.0–1.16	30.0–1.16	30.0–1.16	30.0–1.32	30.0–1.18	30.0–1.70
Used reflections	118,814	117,824	118,833	83,311	113,270	35,393
Occupancy sum of atoms	3,240	3,204	3,262	3,228	3,188	3,120
*R* (%)	12.5	13.1	12.8	14.0	13.0	14.3
*R*_free_ (%)	16.0	17.0	16.0	18.7	17.1	20.7
Rms deviations						
Bond length (Å)	0.016	0.016	0.016	0.012	0.016	0.007
Angle distance (Å)	0.031	0.031	0.032	0.028	0.031	0.025
PDB ID	5Z0D	5Z0E	5Z0F	5Z0G	5Z0H	5Z0M

^a^In the case of ST6, copper-free crystal was directly soaked in a buffer containing CuSO_4_ and NH_2_OH. In the other cases, met2-form crystals were soaked in a buffer containing CuSO_4_ and NH_2_OH.

^b^Values in parentheses are for the highest resolution bin.

^c^*R*_merge_ = ∑|*I* − <*I*>|/∑*I*, where *I* is the observed intensity and <*I*> is the mean value of *I*.

Abbreviations: H63F, complex between the mutated tyrosinase, in which the His^63^ residue is replaced with phenylalanine, and caddie; PDB, Protein Data Bank; Rms, root-mean-square; WT, wild-type complex; Y98F, complex between tyrosinase and the mutated caddie, in which the Tyr^98^ residue is replaced with phenylalanine.

The structure of the oxy-tyrosinase was determined using the crystal of tyrosinase complexed with the caddie Y98F mutant, in which the Tyr^98^ residue is replaced with phenylalanine (ST2 in Table 1). Prior to the data collection, the crystal was soaked in a CuSO_4_-containing solution for 80 h and then in a NH_2_OH-containing solution for 2 h under aerobic conditions at 25 °C. In the structure, electron densities for both Cu^A^ and Cu^B^ are elongated (Fig 3B). In addition, an elongated density, which can be assigned as a peroxide ion, was observed between Cu^A^ and Cu^B^. These findings suggest that the structure represents the mixture of deoxy form (65%)—in which two copper ions are positioned at the Cu^A-1^ and Cu^B-1^ sites—and oxy form (35%)—in which two copper ions are positioned at the Cu^A-2^ and Cu^B-2^ sites (Table 2). We have previously reported an oxy-form structure (PDB ID: 1WX2 at 1.80-Å resolution), which had been prepared by the addition of H_2_O_2_ to the met2-form crystal [12]. However, the structure could not be determined at a high resolution, probably because the crystal had been seriously injured by the reagent. Changing the method for preparation of the oxy form and using the synchrotron radiation made it possible to determine the structure at a high resolution, although the occupancy of peroxide was low. Both Cu^A-2^ and Cu^B-2^ take the monopyramidal tetragonal coordination preferred by Cu(II), similar to the met2 form. The oxy form is in a syn arrangement, in which axial ligands of Cu^A-2^ and Cu^B-2^ are His^63^ and His^216^, respectively. The His^63^ residue is weakly associated to Cu^A-2^, with a long coordination bond distance (2.5 Å).

**Table 2:** Refined occupancies^a^.

	ST1	ST2	ST3	ST4	ST5	ST6
Cu^A-1^	1.00	0.654	0.579	0.542	0.222	-
Cu^A-2^	-	0.346	0.287	0.171	0.359	-
Cu^A-3^	-	-	0.134	0.287	0.418	0.942
Oζ2	-	-	0.035	0.193	0.584	0.300
Peroxide	-	0.346	0.386	0.266	0.194	-

^a^Except the case of ST6, occupancies were refined using the restraints described in the text.

In most of the synthetic models of μ-η^2^:η^2^-peroxo-dicopper(II), the Cu_2_O_2_ core is planar. In contrast, the current oxy form exhibits a bent-butterfly structure in the Cu_2_O_2_ core, where the midpoint between two peroxide oxygen atoms is above the midpoint of Cu^A-2^ and Cu^B-2^ (Fig 3B). Considering that Cu(II) prefers the planar coordination, the bent-butterfly Cu_2_O_2_ structure is less solid than the planar one [3]. The bent structure, which was also observed in the oxy-form structure reported previously [12], has been suggested to be formed by the hydrogen bond interaction between the hydroxyl of the caddie Tyr^98^ and the bridging peroxide. However, the hydroxyl group is absent in the current structure. The tyrosinase may be suitable to take a flexible bent-butterfly structure in the oxy form to allow the conformational change during the reaction, as proposed by the other research group [24].

To visualize the process in the tyrosinase reaction toward the caddie Tyr^98^ residue, we determined the crystal structures, each of which was cryo-trapped after the aerobic soaking of the crystal of met2-tyrosinase complexed with the caddie protein in a buffer containing CuSO_4_ and NH_2_OH for a given time at 25 °C (ST3 to ST5 in Table 1). After soaking the crystal for 10 min at 25 °C (ST3), the electron density map indicates the coexistence of the deoxy and the oxy forms in the crystal (Fig 3C), as observed in ST2. The anomalous difference Fourier map suggests another copper-binding site, which is approximately equidistant from Cu^A-2^ and the hydroxyl of the caddie Tyr^98^, although the electron density is very weak (Fig 3C). This is in striking contrast to the results obtained from ST2, at which the anomalous difference Fourier map and *F*_o_-*F*_c_ map did not show any signal at that position. The density at this site becomes strong in the other structures, as described below. Additionally, using the diffraction data of another crystal collected at the wavelengths of 1.35 and 1.40 Å, we confirmed that the position was occupied by the copper atom. Hereafter, we refer to copper observed at the new position as Cu^A-3^. The information on the important distances in the two possible oxy-form structures is shown in S1B and S1C Fig. At the Cu^A-3^ position, a new coordination bond is formed with the hydroxyl of the caddie Tyr^98^ residue, whereas the coordination bond with the His^63^ residue is completely lost. In ST3, occupancies of Cu^A-1^, Cu^A-2^, and Cu^A-3^ were calculated to be about 0.6, 0.3, and 0.1, respectively, whereas occupancies of Cu^B-1^ and Cu^B-2^ were about 0.6 and 0.4, respectively (Table 2).

**Fig 4:**
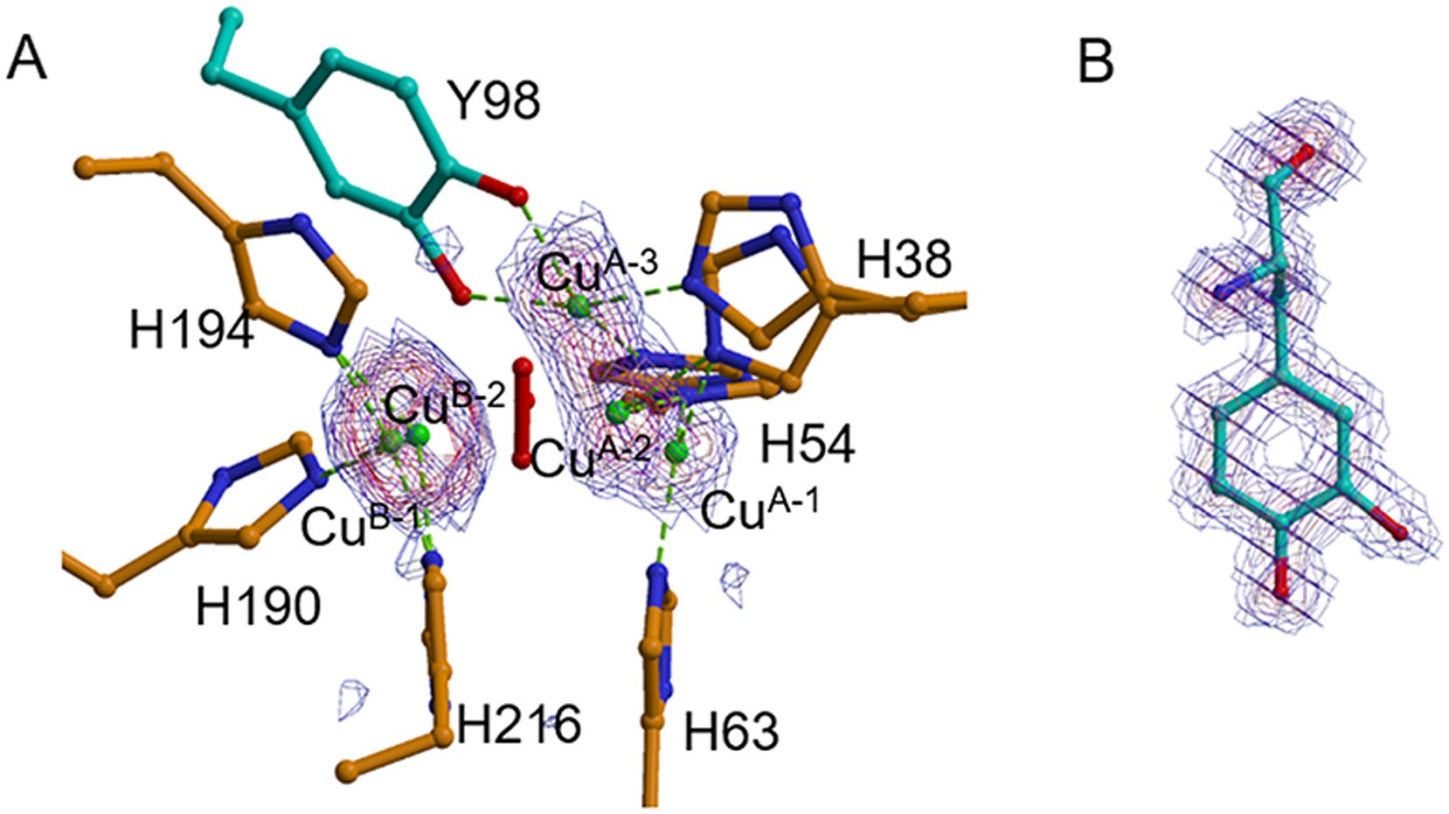
Maps around the dicopper center in tyrosinase. (A) Anomalous difference Fourier map in ST5 contoured at 3 σ, starting at 3 σ. Anomalous data collected at the wavelength of 0.9 Å are sufficient to determine the positions of copper atoms because the *f”* value at 0.9 Å is high (1.9 e), even in comparison with that at the peak wavelength (3.9 e at 1.38 Å). (B) *F*_o_−*F*_c_ omit map in ST5, which was calculated based on a hypothesis that modified Tyr^98^ residue is not contained in the model. The map was contoured at 3 σ, starting at 3 σ. H, histidine; Y, tyrosine.

In the crystal structures obtained at 20 min (ST4) and 2 h (ST5) after the aerobic addition of NH_2_OH, the electron density at the Cu^A-3^ site is stronger than that obtained at 10 min (Fig 3D and 3E). The high occupancy of Cu^A-3^ was also suggested by the anomalous difference Fourier map (Fig 4A). The electron density maps also indicate that the side chains of the His^38^ and His^54^ residues clearly take two different conformations. One conformation is suitable for the coordination to Cu^A-1^ and Cu^A-2^ and, the other is suitable for the coordination to Cu^A-3^. Although the flexibility of the His^54^ residue was recognized by early studies [12,26], the His^38^ residue also has the flexibility to adapt to the movement of Cu^A^. The flexibility of the His^38^ residue is enabled by the removal of Wat^1^.

In ST5, occupancies of Cu^A-1^, Cu^A-2^, and Cu^A-3^ were calculated to be about 0.2, 0.4, and 0.4, respectively, whereas occupancies of Cu^B-1^ and Cu^B-2^ were about 0.2 and 0.8, respectively (Table 2). This result implies that, when Cu^A^ occupies the Cu^A-3^ site, Cu^B^ is positioned at the Cu^B-2^ site. The electron density at the bridging position between the two copper ions is also elongated (Fig 3E), indicating the heterogeneity at this site. The major bridging molecule (80%) seems to correspond with a molecule containing one oxygen atom (water or hydroxide ion) positioned at the Wat^3^ site. The minor molecule (20%) seems to be peroxide, although the binding mode is different from that in the above-mentioned oxy forms (Fig 3B to 3D). In detail, one oxygen atom in the peroxide exists at a different position, where it can form a hydrogen bond with the Nε atom of the His^63^ residue (S1D Fig).

**Fig 5:**
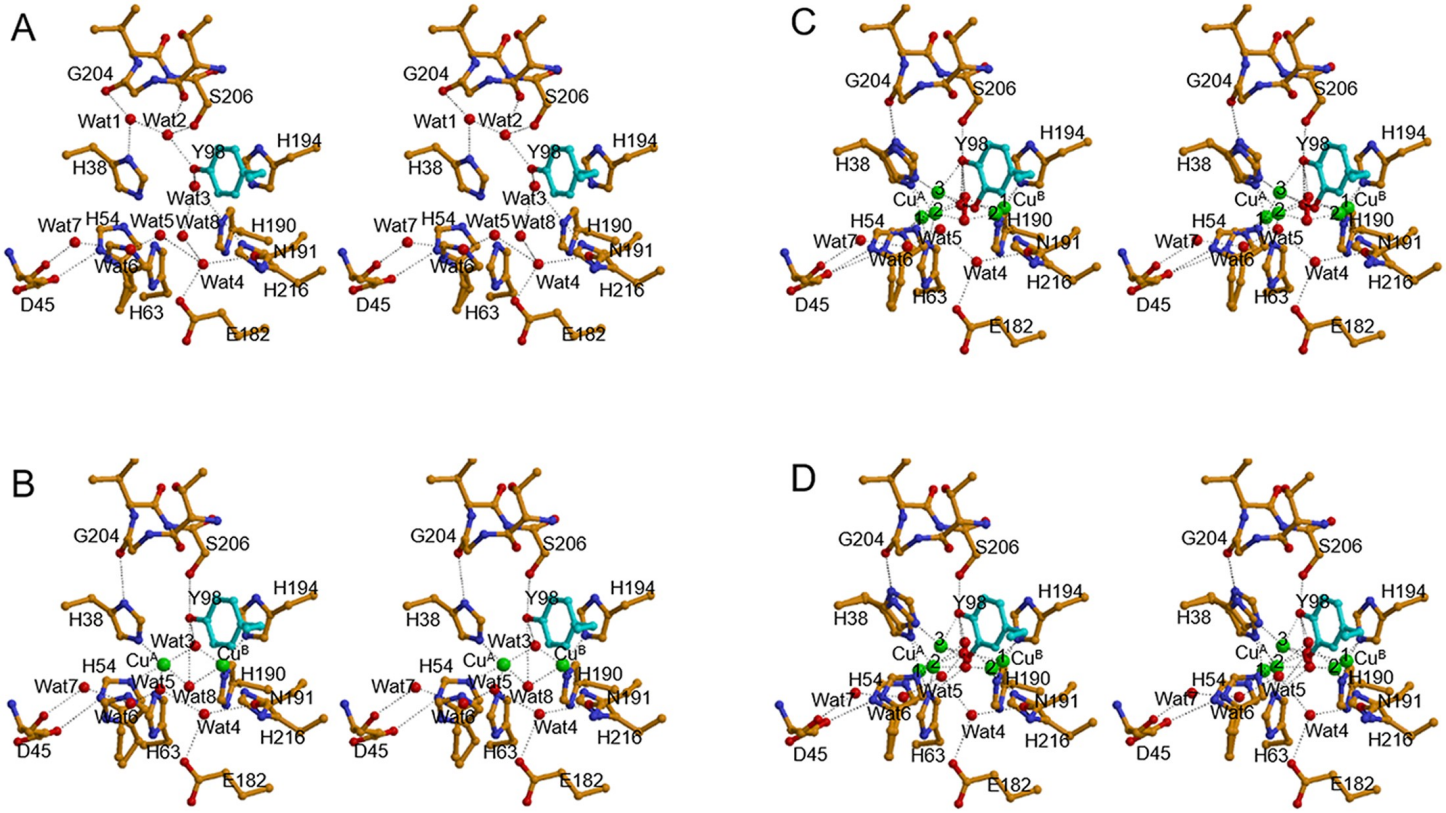
Structural models around the catalytic site of tyrosinase complexed with the caddie protein. (A) Cu(II)-free form. (B) met2 form. (C) ST3. (D) ST5. Wat^5^, Wat^6^, and Wat^8^ are not completely occupied in (A), whereas Wat^5^ and Wat^6^ are not in (B). In (C) and (D), peroxide and Wat^3^ are present between Cu^A^ and Cu^B^. D, aspartic acid; E, glutamic acid; G, glycine; H, histidine; N, asparagine; S, serine; Wat, water molecule; Y, tyrosine.

Furthermore, in ST5, clear electron densities were found around the Cε2 atom of the caddie Tyr^98^ residue (Figs 3E and 4B), indicating that the reaction proceeded even in the crystalline state. The density in this case corresponds to an oxygen atom with the occupancy of about 0.6 (Table 2). The newly added oxygen (Oζ2) is within the coordination bond distance from Cu^A-3^ and near to Cu^B-2^ (S1E Fig). The complex between Cu^A-3^ and the oxygenated Tyr^98^ may correspond with the Cu(II)-bound dopasemiquinone observed in the solution state [29]. Specifically, Cu^A-3^ is in a bipyramidal trigonal coordination cage, in which the axial ligands are the Oζ2 atom added to the caddie Tyr^98^ and the Nε atom of His^38^, and equatorial ligands are the Oη atom of the caddie Tyr^98^, the Nε atom of His^54^, and the bridging oxygen atom at the Wat^3^ site. The Nε atom of His^63^ is not coordinated with Cu^A^, but it is within the hydrogen-bonding distance of one of the peroxide oxygens (2.8 Å) and Wat^3^ (3.4 Å) (S1D and S1E Fig). The structural refinement suggests that the occupancies of Cu^A-2^ and Cu^B-2^ are higher than the values obtained at the earlier times (ST3 and ST4), whereas the occupancy of peroxide is lower (Table 2). Therefore, in ST5, a large part of Cu^B-2^, as well as a part of Cu^A-2^, seems to take tetrahedral coordination, which is preferred by Cu(I), with three Nε atoms from the histidine residues and one oxygen molecule at the Wat^3^ site in each (Cu^B-2^ in S1E Fig and both Cu^A-2^ and Cu^B-2^ in S1F Fig). Atomic models of the active site in ST3 and ST5 are shown in Fig 5C and 5D, respectively, together with those in the Cu(II)-free form (Fig 5A) and the met2 form (Fig 5B).

In crystallography, refinements of both the occupancy and the temperature factors of atoms are difficult. In the present case, each crystal is considered to contain intermediates in a different ratio. Therefore, occupancies were refined using the following restraints. When Cu^A^ occupies the Cu^A-1^, Cu^A-2^, and Cu^A-3^ sites, Cu^B^ is likely positioned at the Cu^B-1^, Cu^B-2^, and Cu^B-2^ sites, respectively. In addition, when Cu^A^ occupies the Cu^A-3^ site, the His^38^ and His^54^ residues seem to take the minor conformations. Therefore, the occupancy of Cu^A-1^ was set to equal that of Cu^B-1^, and the sum of occupancies of Cu^A-2^ and Cu^A-3^ was set to equal that of Cu^B-2^. The occupancies of the major and minor conformations of the His^38^ and His^54^ residues were set to equal the sum of occupancies of Cu^A-1^ and Cu^A-2^ and the occupancy of Cu^A-3^, respectively. The hydroxylation reaction must proceed after the binding of oxygen to the deoxy form, where the Wat^3^ atom is positioned between the Cu^A-1^ and Cu^B-1^ sites. However, after the oxygenation, one of the peroxide oxygens is attached to the Cε2 atom of the Tyr^98^ residue, whereas the other oxygen seems to occupy the Wat^3^ site. Therefore, the sum of occupancies of peroxide and the Oζ2 atom added to Tyr^98^ was also set to equal that of Cu^B-2^, and the sum of occupancies of peroxide and one oxygen atom at the Wat^3^ site was set to equal 1. Temperature factors of a copper ion and its ligands were refined to become similar values using DELU and SIMU restraints in the SHELXL-97 program [43]. Refined occupancies and equivalent *B*-factors are shown in Table 2 and S1 Table, respectively. In ST3 and ST4, the occupancy of the Oζ2 atom is lower than that of Cu^A-3^, whereas in ST5, the occupancy of the Oζ2 atom is higher than that of Cu^A-3^. These observations suggest that Cu^A^ moves to the Cu^A-3^ site prior to the oxygenation reaction, whereas Cu^A^ moves back to the Cu^A-2^ site after the reaction.

To investigate whether the movement of Cu^A^ between the Cu^A-2^ and the Cu^A-3^ sites is important for the catalytic reaction, a complex between the H63F-mutated tyrosinase, in which the His^63^ residue is replaced with phenylalanine, and the caddie protein was prepared. The protein complex did not form the aggregate of the caddie protein after the addition of CuSO_4_ and NH_2_OH. However, the spectroscopic analysis suggests that the Cu(II)-bound semiquinone complex is formed under the alkaline condition at pH 9, but the formation rate is slow. In this case, the met2 form was unable to be generated in the crystal even after longer incubation with Cu(II), which is sufficient for the wild-type crystal to generate the met2 form, probably because of the defect at the Cu^A^-binding site. Soaking in a buffer containing CuSO_4_ and NH_2_OH was necessary to generate the dicopper center. The crystal structure of the H63F-mutated complex was obtained by using a crystal aerobically soaked in a buffer containing CuSO_4_ and NH_2_OH for 24 h at 25 °C (ST6 in Table 1). At the active center in the H63F-mutated tyrosinase, Cu^A^ is found at one site. The distances between the site in ST6 and Cu^A-1^, Cu^A-2^, or Cu^A-3^ sites in ST5 are 2.7, 1.9 or 0.2 Å, respectively. Cu^A^ is localized at the Cu^A-3^ site probably because of the mutation at the His^63^ residue, which is a ligand of Cu^A-1^ and Cu^A-2^. Based on the temperature factors (S1 Table), the Cu^A-3^ atom and the His^54^ residue are unstable. However, Cu^B^ is found at either the Cu^B-1^ or the Cu^B-2^ site. In contrast to the wild-type complex, the position of Cu^B^ seems to be variable when Cu^A^ occupies the Cu^A-3^ site. The positional uncertainty of Cu^B^ may depend on the structural uncertainty at the Tyr^98^ residue, although the correct reason is unknown at this time. The sum of occupancies of Cu^B-1^ and Cu^B-2^ was calculated to be approximately 1.0, whereas the occupancy of Cu^A-3^ was approximately 0.9 (Table 2), suggesting the slight incompleteness of the copper uptake. On the other hand, although a part of the caddie Tyr^98^ residues seems to be converted to dopasemiquinone, the occupancy of the Oζ2 atom was calculated to be 0.30 (Table 2). In this case, a part of Cu^A-3^ is ligated to the oxygenated Tyr^98^ residue, whereas the rest is ligated to the unmodified one. This is similar to the results obtained using the wild-type crystal at the early stage, as the occupancy of the Oζ2 atom is lower than that of Cu^A-3^, indicating that the movement of Cu^A^ to the Cu^A-3^ site occurs prior to the hydroxylation reaction. In addition, it is thought that Cu^A^ was unable to move to the Cu^A-2^ site after the reaction because of the impairment of the site, which might inhibit the progress of the reaction toward dopaquinone and thereby inhibit the aggregation of the caddie protein.

## Discussion

In the previous study [29], we demonstrated that the addition of NH_2_OH stimulates the caddie proteins to aggregate, resulting in the release of tyrosinase from the complex. The aggregation is likely triggered by the formation of reactive dopaquinone on the caddie Tyr^98^ residue. The UV-vis and resonance Raman spectroscopic analyses indicate that the Tyr^98^ residue is converted to a reactive quinone through the formations of the μ-η^2^:η^2^-peroxo-dicopper(II) and Cu(II)-dopasemiquinone intermediates. It is important to note that intermediates after the μ-η^2^:η^2^-peroxo-dicopper(II) generation have not been trapped when adding the poor substrate (3,5-difluorophenol) to the *S*. *antibioticus* tyrosinase [44], which shares high similarities with the *S*. *castaneoglobisporus* tyrosinase used in the present study. The caddie Tyr^98^ residue may be a poorer substrate than 3,5-difluorophenol, enabling detection of the intermediates.

After the crystal structure of tyrosinase was shown by our group [12], its catalytic mechanism has been actively discussed by other groups [4,6,15,23,24,27,28,30–33,40–42]. Since tyrosinase can react with the Tyr^98^ residue of the caddie protein [29], the Tyr^98^ residue is expected to adopt a similar binding position of L-tyrosine as a genuine substrate of tyrosinase. However, free L-tyrosine, which lacks the structural restraints as compared with the Tyr^98^ residue as a part of the caddie protein, may be bound deeply into the active-site pocket. The hydroxyl group of the caddie Tyr^98^ residue interacts with the hydroxyl group of Ser^206^, and the phenol ring has a stacking interaction with the imidazole ring of His^194^ (Fig 2B). Therefore, when a genuine substrate is bound to the active center of the *Streptomyces* tyrosinase, it must interact with Ser^206^ and His^194^. The computer simulation analysis also suggests that the interactions with Ser^206^ and His^194^ are important for the binding of kojic acid, which is a tyrosinase inhibitor, to the active center of the *Streptomyces* tyrosinase [42]. However, the Ser^206^ residue is not conserved in tyrosinases from other microorganisms.

As the first step in the hydroxylation reaction of tyrosinase, in general, the substrate hydroxyl was assumed to bind directly to one of the two copper ions in the oxy form. However, since the hydroxyl group of the Tyr^98^ residue has no direct interaction with the copper atoms in the starting oxy form, the movement of the Tyr^98^ residue and/or the structural change of the active center of tyrosinase must occur prior to the hydroxylation reaction. Another research group has already anticipated that genuine substrate binds to tyrosinase in a manner similar to the caddie Tyr^98^ residue [4,30]. They insisted that the substrate must shift toward Cu^A-2^ from the position of the caddie Tyr^98^ residue to form a coordination bond. In the crystal structure of the *Bacillus* tyrosinase complexed with tyrosol (PDB ID: 4P6T) [31], the substrate was found at the position about 2 Å from that of the caddie Tyr^98^ residue, and the hydroxyl is bound at the axial position of Cu^A^. Additionally, in the case of the B subunit, Cu^A^ has a very long coordination distance with the residue corresponding to His^63^ upon the binding of the substrate. Because of positional restriction by the surrounding residues, the binding position of the caddie Tyr^98^ residue may be slightly different from that of the small substrate, resulting in emphasis of the importance of the Cu^A^ movement. In addition, our crystallographic results (ST3, ST4, and ST6) indicated that the movement of Cu^A^ to the Cu^A-3^ site occurs prior to the hydroxylation reaction. In total, Cu^A-3^ seems to be the functional site but not the artifact one occupied only in the product-bound state.

**Fig 6:**
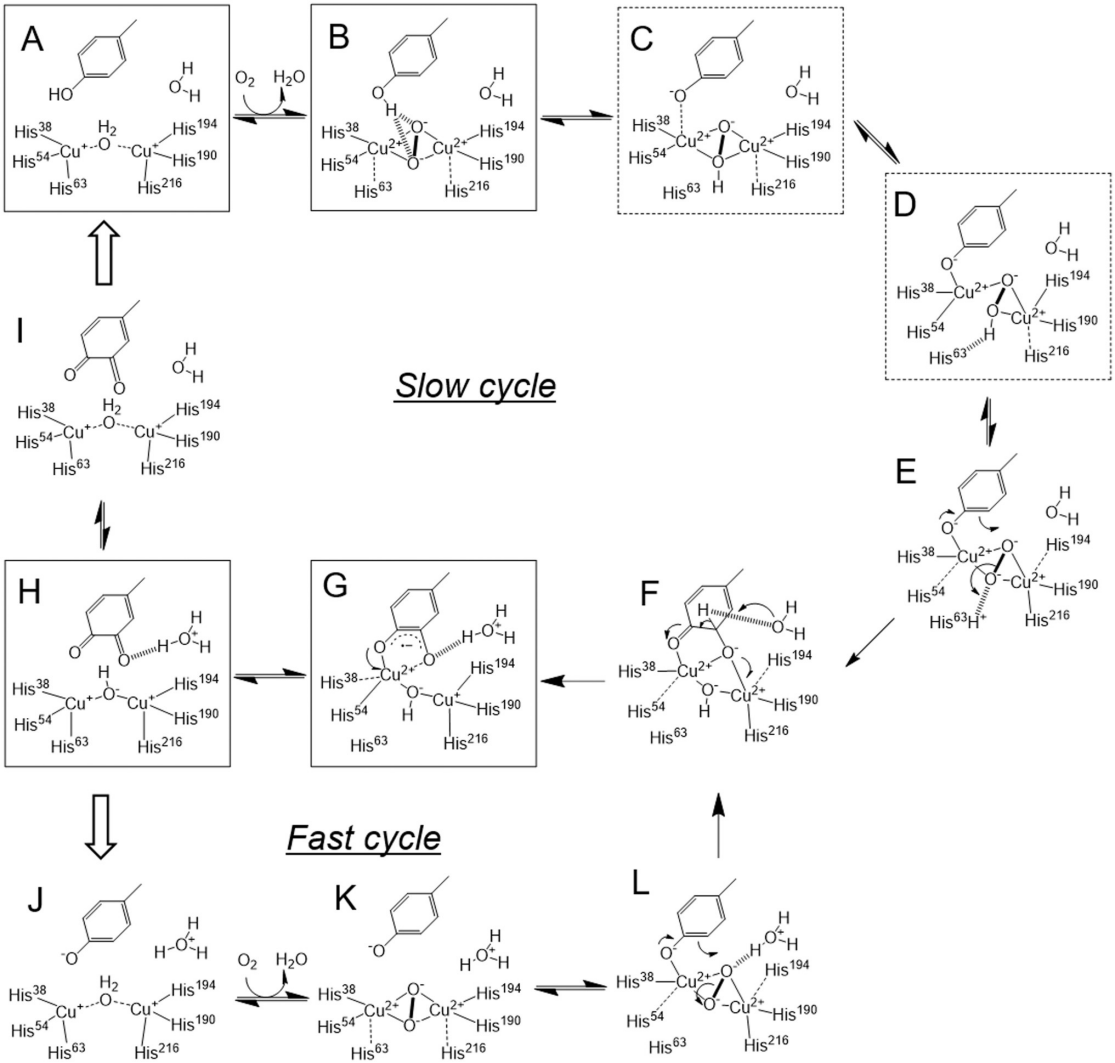
Proposed catalytic mechanism of tyrosinase toward the caddie Tyr^98^ residue. Detailed explanations are given in the text. (A), (B), and (C) intermediates are likely to be found at the early stage, whereas (D), (G), and (H) are likely to be found at the late stage. The steps including the release of product and the incorporation of new substrate are indicated by the outlined arrows.

From the current data, we propose a catalytic mechanism of tyrosinase toward the caddie Tyr^98^ residue as shown in Fig 6. In the deoxy form as a starting point (Fig 6A), two copper ions are located at the Cu^A-1^ and Cu^B-1^ sites. When the dioxygen is bound to the deoxy form, Cu^A^ and Cu^B^ move toward the Cu^A-2^ and Cu^B-2^ sites, respectively. The movement is triggered by the bonding interactions between dioxygen and Cu(I) atoms, together with the change in oxidation states from Cu(I) (which prefers trigonal or tetrahedral coordination) to Cu(II) (which prefers tetragonal or monopyramidal tetragonal coordination). In the oxy form, two oxygens of dioxygen are positioned at the Wat^3^ and Wat^8^ sites, resulting in the formation of a μ-η^2^:η^2^-peroxo-dicopper(II) with a bent-butterfly structure (Fig 6B).

Deprotonation of the substrate hydroxyl is thought to be important for the hydroxylation reaction of tyrosinase [34]. Based on the observation that hydroxylation of the caddie Tyr^98^ does occur in the crystalline state at pH 6.5, a base must be present in proximity to the hydroxyl group. The base that deprotonates the hydroxyl has been under debate [4,6,12,15,23,24,27,28,31,33,40]. Although the recent study suggests that Wat^4^, which forms hydrogen bonds with the Glu^182^ and Asn^191^ residues, acts as a base [6,15,23,28,31], it is far away from the hydroxyl oxygen of the caddie Tyr^98^ residue (5.9 Å) (S1B Fig). Additionally, in contrast to the cases of large tyrosinases, since the substrate-binding pocket of the small *Streptomyces* tyrosinase is directly exposed to the solvent region in the absence of the caddie protein (Fig 2A), there may be no different binding positions for the substrate. Therefore, deprotonation of the substrate is unlikely to occur at the entrance or during the preorientation by the second-shell residues located near the active site, as proposed by other groups [15,23,33]. In the crystal structure of oxy-tyrosinase complexed with the caddie protein, the hydroxyl group is positioned near the two peroxide oxygens within a distance of 3.5 Å (S1B Fig). This indicates that the hydroxyl proton can easily move to the peroxide. Studies using model systems with low molecular weight [4,34] suggest that the neutral substrate is difficult to be hydroxylated. The difficulty is explained as follows: after the binding of the neutral substrate to the dicopper center and the subsequent transfer of a proton from the substrate to peroxide, one electron is transferred from the substrate to one of the two copper ions, leading to the formation of C–C coupled dimer products, like a tyrosine dimer. Considering from a different perspective, protonation of the dicopper center may diminish or largely decrease the hydroxylation activity, which affords the side reaction to generate the C–C bond. On the other hand, tyrosinase has not been reported to generate the C–C coupled dimer, probably because of the high reaction rate of the enzyme or of the situation of the substrate in the active-site pocket, which prevents the dimer formation.

It should be noted that a coordination bond between Cu^A^ and His^63^ is completely lost after the movement of Cu^A^ to the Cu^A-3^ site and that the distances between the peroxide oxygens and the Nε atom of His^63^ are in the range of 3.5 and 4.0 Å (S1B Fig). This histidine flexibility opens the opportunity for the imidazole to serve as a base to deprotonate the phenol substrate. Additionally, a recent study using a small-molecule model suggests that the copper ligand acts as an internal base for the substrate hydroxyl, since the addition of excess copper ligand enables the oxygenase reaction toward protonated phenol [45]. Therefore, we assume that a proton from the Tyr^98^ hydroxyl moves to the His^63^ residue via the peroxide. Although there is another possibility that the proton moved to Wat^4^ via the peroxide, the distance between one of the peroxide oxygens and the Wat^4^ atom is slightly larger (4.2 Å) than the distance to the Nε atom of His^63^. The significance of the proton transfer step is partially supported by the results using the H63F-mutated complex. The mutant protein does not actually have reactivity toward the small substrate (L-tyrosine), even under the condition in which the dicopper center could be formed, and the reaction toward the caddie Tyr^98^ residue was arrested at the Cu(II)-dopasemiquinone intermediate, suggesting that the reaction catalyzed by the mutant is halted at the first turnover. In ST6, the occupancy of the oxygen atom added to the Tyr^98^ residue is significantly lower than that of Cu^A-3^. In this case, probably because of the lack of the internal base, a large part of the hydroperoxide ion, which was formed between two coppers after movement of the proton from the Tyr^98^ hydroxyl, would be replaced by a water or hydroxide ion prior to the deprotonation to produce peroxide. However, when the Tyr^98^ residue was deprotonated beforehand, hydroxylation reaction could proceed. This hypothesis is supported by the observation that the alkaline pH conditions stimulate the change in the UV-vis spectrum, which indicates the accumulation of Cu(II)-dopasemiquinone intermediate. In addition, this result may exclude the possibility that (μ-oxo)(μ-hydroxo)-dicopper(II,III) acts as an active species for the hydroxylation, which was proposed from the simulation analysis [40], at least in our system.

The deprotonation of the hydroxyl seems to have two roles. At first, since the ortho-carbon has a partial negative charge after the deprotonation of the hydroxyl, the atom comes to exhibit a high nucleophilicity. An electrophilic aromatic substitution reaction by the oxy form has been suggested for the hydroxylation mechanism on tyrosinase [34]. The deprotonation of the substrate hydroxyl may also play a role in generating an electrostatic interaction between Cu^A^ and the hydroxylate, which induces the movement of Cu^A^ to the Cu^A-3^ site (about 1.7 Å). The large movement is not surprising given that the large structural changes are observed to create the side-on peroxide species from the reaction of the reduced enzyme and dioxygen (1.0 and 0.5 Å for Cu^A^ and Cu^B^, respectively). The location of Cu^B^ is better conserved than that of Cu^A^, which is in agreement with the previous observations [12,26,31] as well as with the recently elucidated crystal structure of Zn(II)-bound TRP1 [25]. The energetic driving force for the movement may be placing the strongest sigma-donating ligand (phenolate) into an equatorial position. Together with the movement of Cu^A^, the side chains of His^38^ and His^54^ change conformation to maintain the coordination bond with Cu^A^. The movement of Cu^A^ is also found in the crystal structure of the *Bacillus* tyrosinase complexed with tyrosol [31] and in the simulated structure of the *Streptomyces* tyrosinase complexed with kojic acid [42], although the movement lengths are shorter than that observed in the present study.

In accordance with the movement of Cu^A^, peroxide must move to a new position. Although the crystal structure is absent, we propose a hypothesis that the peroxide is arranged keeping the μ-η^2^:η^2^-binding mode (Fig 6E), which may be useful to destabilize the O−O bond for the reaction. In this putative intermediate, two copper ions are positioned at the Cu^A-3^ and Cu^B-2^ sites, one of two peroxide oxygens (O^proximal^) is near the ortho-position of the caddie Tyr^98^ residue, and the other (O^distal^) is at the Wat^3^ site. The Cu_2_O_2_ core lies on a plane created by the Oη atom of the caddie Tyr^98^ and the Nε atoms of His^38^, His^190^, and His^216^. This intermediate is in an anti-arrangement in which the axial ligands of Cu^A^ and Cu^B^ are His^54^ and His^194^, respectively. Our previous crystallographic studies [12,26] have indicated the flexibility of His^54^. In addition, the distance from Cu^B-2^ to the Nε atom of His^216^ is comparable with that to the Nε atom of His^194^ and is longer than that to the Nε atom of His^190^ (S1B to S1F Fig). These observations suggest that the axial-to-equatorial exchange of His^216^ and the equatorial-to-axial exchanges of His^54^ and His^194^ would occur. As possible intermediates between Fig 6B and 6E, we propose two structures based on the crystal structures (S1C and S1D Fig), since the different binding modes of peroxide are present in the early stage (ST3 and ST4) and the late stage (ST5). In the first structure (Fig 6C), two copper ions are positioned at the Cu^A-3^ and Cu^B-2^ sites, and peroxide is positioned at the original site, leading to the formation of reverse butterfly structure. The proton from the caddie Tyr^98^ residue may be attached to peroxide. In the second one (Fig 6D), two copper ions are positioned at the Cu^A-3^ and Cu^B-2^ sites, and peroxide is positioned at the site observed in the later stage. The proton attached to peroxide may interact with the Nε atom of the His^63^ residue.

Orientation of the substrate to the dicopper center is crucially important for the tyrosinase reactivity. For the hydroxylation reaction, the σ* orbital of the bridging peroxide ligand must overlap with the π orbitals of the substrate. To this end, rotation of the peroxide [4,30], rotation of the substrate [31], and axial-to-equatorial interchanges of the His^54^ and His^63^ residues [41] were proposed to occur after the binding of the substrate hydroxylate to Cu^A^. In the present study, we propose that the orientation of the substrate is adjusted by the movement of Cu^A^, which accompanies the syn-to-anti rearrangement of the copper ligands. By the rearrangement, the hydroxyl group of the Tyr^98^ residue was changed to an equatorial ligand of Cu^A^. After the adjustment, O^proximal^ attacks the ortho-carbon of the caddie Tyr^98^ residue electrophilically. The reaction would be followed by the cleavage of the O–O bond and the proton transfer from His^63^ to the oxide ion derived from O^distal^, resulting in the formation of a dienone intermediate with a nonplanar ring (Fig 6F).

The axial-to-equatorial exchange of the substrate hydroxylate was previously proposed for the reaction mechanism of the tyrosinase model with a low molecular weight, in which the μ-η^2^:η^2^-peroxo-dicopper(II) catalyzes the conversion of a deprotonated phenol to the Cu(II)-bound semiquinone [35,36,39]. The current study presents the first evidence that the axial-to-equatorial exchange actually occurs in the macromolecular system. The research group demonstrated that an axial-to-equatorial reorientation of the substrate hydroxylate induces cleavage of the O–O bond prior to the oxygenation reaction, resulting in the generation of bis(μ-oxo)-dicopper(III), which has an absorption peak at about 400 nm because of the oxide-to-Cu(III) charge transfer transition [35,36,39]. However, our previous study of the solution state indicated that after the development of the oxy form with a μ-η^2^:η^2^-peroxo-dicopper(II) core, the Tyr^98^ residue of the caddie is converted to dopaquinone via Cu(II)-bound dopasemiquinone [29]. Since Cu(II)-dopasemiquinone and intact dopaquinone also have an absorption peak at about 400 nm, it is difficult to confirm the generation of the cleaved species from the UV-vis spectrum. Furthermore, we could not detect any cleaved species, such as bis(μ-oxo)-dicopper(III), although resonance Raman analysis using a 413-nm laser was conducted intensively under the different temperature conditions [29]. However, since the lifetime of the cleaved species may be too short for detection, we cannot exclude the possibility that O–O bond scission occurs prior to the reaction.

Based on the results obtained in the solution state [29], the crystal structures prepared by soaking for a longer time may contain Cu(II)-dopasemiquinone or dopaquinone in high ratios. In the proposed dopasemiquinone-bound structure (Fig 6G), Cu(II) and Cu(I) are present at the Cu^A-3^ and Cu^B-2^ sites, respectively. Hereafter, we refer to the structure bound with dopasemiquinone as a half-met form. Cu^A-3^ takes the bipyramidal trigonal coordination preferred by Cu(II) next to the tetragonal or monopyramidal tetragonal coordination, whereas Cu^B-2^ takes the tetrahedral coordination preferred by Cu(I). The bipyramidal trigonal coordination was also detected in early studies using a half-met form of the *Neurospora* tyrosinase complexed with the tyrosinase inhibitors such as mimosine or benzoic acid [2,46,47], although the active site of the *Neurospora* tyrosinase is different from that of the *Streptomyces* tyrosinase because of the presence of the cysteine–histidine thioether bond. The η^2^-semiquinone-bound half-met form may be formed via the η^2^:η^1^-catecholate-bound met form with a one-electron reduction of Cu^B^. The η^2^:η^1^-catecholate-bound dicopper(II) was previously proposed as an intermediate of the tyrosinase reaction [35–37,48–50]. However, if the η^2^:η^1^-catecholate-bound intermediate was formed in our system, the ring of the caddie Tyr^98^ would become unparallel to the ring of the tyrosinase His^194^, generating the steric hindrance. Therefore, the deprotonation of the ortho-carbon in the nonplanar intermediate (Fig 6F to 6G) may be coupled with the one-electron reduction of Cu^B^ to avoid the formation of the undesirable η^2^:η^1^-catecholate-bound intermediate.

For the formation of the G intermediate, another base to abstract a proton from the ortho-carbon of the substrate is needed. Although the deprotonation of the substrate hydroxyl is recognized as an important step, the significance of the base at the second deprotonation step seems to be underestimated by many research groups. As a consensus, the proton is thought to finally move to oxide or hydroxide ion derived from O^distal^, resulting in the conversion to a hydroxide ion or a water molecule, respectively. Direct movement of the ortho-carbon proton to the O^distal^-derived oxygen is unlikely, since the O^distal^-derived atom and the atom added to the substrate are on the same side with respect to the phenol ring. This step has been proposed to be mediated by a base such as His^54^ [40] or a solvent atom [36]. Since the Nε atom of His^54^ is distant from the Cε2 atom of the caddie Tyr^98^ (4.2 Å, S1E Fig), the residue is unlikely to act as a base. Similarly, Wat^4^, which was recently considered as a hopeful base by other groups [6,15,23,28,31], is also distant from the Cε2 atom of the caddie Tyr^98^ (4.1 Å). However, adjacent Wat^5^ is closer to the Cε2 atom (3.6 Å). Therefore, we now consider Wat^5^ as a candidate base at this step. The generated hydroxonium ion at the Wat^5^ site may be stabilized by the hydrogen-bonding network to the well-conserved Glu^182^ residue via Wat^4^.

Our crystallographic results indicate that Cu^A^ moves back to the Cu^A-2^ position after the oxygenation reaction. The movement may be coupled with the one-electron transfer from semiquinone to Cu^A^, resulting in the putative complex between the deoxy form and dopaquinone (Fig 6H). Hereafter, we refer to the structure bound with dopaquinone as a deoxy2 form, in which two cuprous ions are positioned at the Cu^A-2^ and Cu^B-2^ sites. Both Cu^A-2^ and Cu^B-2^ take the tetrahedral coordination preferred by Cu(I) (S1F Fig). The deoxy2 form has a shorter Cu–Cu distance than the starting deoxy form (Fig 6A). This may be due to the difference in bridging molecule. That is, deoxy and deoxy2 forms have a water molecule and a hydroxide ion at the bridging position, respectively. Movement of the proton from Wat^5^ to the O^distal^-derived oxygen may result in the formation of deoxy form (Fig 6I).

The aggregation of the caddie protein seems to progress through the generation of intermolecular linkage between the liberated molecules after the quinone formation. As shown in the previous study [29], the aggregation of the caddie protein was stimulated under the acidic pH conditions probably because of the induction of the release of the caddie from the complex. Since the acidic pH condition rather weakens the linking reaction, the release of the caddie from the complex may be the rate-limiting step for the aggregation. The conversion from the dopaquinone-bound deoxy2 form (Fig 6H) to the deoxy form (Fig 6I) would be stimulated under the acidic pH conditions. The resulting deoxy form may interact with the dopaquinone residue more weakly than the deoxy2 form because of the shift of the copper positions, inducing the release of the quinone-containing caddie protein.

In the catalytic mechanism of tyrosinase generally accepted, the product quinone is separated from the deoxy form, and a new substrate enters the binding pocket. However, the reaction mechanism of tyrosinase in the catalytic cycle may be different from that in the first cycle, as suggested by another group [4]. In this case, it might be unnecessary to develop the deoxy form. That is, the product quinone bound to the hydroxide-bridged deoxy2 form (Fig 6H) is replaced by the next substrate. Then, the bridged hydroxide deprotonates the substrate hydroxyl. The deprotonated substrate is bound to the active center of tyrosinase (Fig 6J) prior to the creation of the oxy form (Fig 6K). If so, the hydroxylation reaction may progress quickly without bases (Fig 6L to 6F), which are currently assumed to be peroxide and His^63^. In the present proposition, the hydroxonium ion at the Wat^5^ site, generated after the second deprotonation step (Fig 6F to 6G), may be stabilized by the interaction with the conserved Glu^182^ residue via Wat^4^. The stabilization effect may inhibit the proton transfer to the O^distal^-derived hydroxide ion and thereby the development of the deoxy form, although it might be not enough to inhibit the transfer to the oxide ion (Fig 6L to 6F). The basicity of Wat^4^ has recently been considered to be a structural factor to distinguish tyrosinase from catechol oxidase [6,15,23,28,31] rather than the accessibility of the substrate to the active center [2,4,12,51,52]. The stabilization of the H intermediate containing a hydroxonium ion may be an important factor to distinguish tyrosinase from catechol oxidase. That is, tyrosinase can adopt the fast catalytic cycle via the deoxy2 form, whereas catechol oxidase only adopts the slow catalytic cycle via the deoxy form, resulting in the apparent low reactivity toward the phenol compound. This proposition must be verified by the various approaches.

In summary, we can propose the chemically reasonable atomistic postulate of the reaction mechanism of tyrosinase toward the caddie Tyr^98^ residue, in which the coordination preference determined by the oxidation state of copper and the protonation state triggers the generation of reaction intermediates in order. However, the binding position of the caddie Tyr^98^ residue may be different from that of a genuine substrate. As proposed by other research groups [4,30,31,41], the hydroxyl group of a substrate is likely bound to the axial position of Cu^A^ in the oxy form (Cu^A-2^). Although the proposition seems to be right for the small substrate, we believe that an important step for the tyrosinase reaction is a subsequent syn-to-anti rearrangement of copper ligands. As a result, substrate hydroxyl is bound to the equatorial position of Cu^A^, and peroxide is optimally repositioned to attack the substrate. This rearrangement is enabled by the movement of Cu^A^ and the scission of a coordination bond between Cu^A^ and His^63^. The migration length of Cu^A^ for the hydroxylation of a genuine substrate may be shorter than that for the hydroxylation of the caddie Tyr^98^ residue. The hydroxylation reaction would accelerate with the decrease in migration length of Cu^A^. In addition, in the binding position of a small substrate, Wat^4^ may be close to the peroxide and enable deprotonation from the hydroxyl group of the caddie Tyr^98^ residue via peroxide. Similarly, His^54^ or Wat^4^ may be close to the ortho-carbon of the substrate, enabling deprotonation at the second step. Identification of the actual bases for the small substrate is the next objective to be pursued.

## Methods

### Mutation

To introduce an H63F mutation in tyrosinase, a QuikChange Site-Directed Mutagenesis Kit (Stratagene) was used. pET-*tyrC* [11], a plasmid for the expression of the His_6_-tagged tyrosinase, was amplified using sense primer (5′-CGTTCCTGCCCTGGTTCCGCAGATTCCTG-3′, where underline means the mutation site) and antisense primer (5′-CAGGAATCTGCGGAACCAGGGCAGGAACG-3′). The original plasmid was removed by DpnI digestion. The mutant plasmid was amplified in the *Escherichia coli* cells, and the introduction of the mutation was confirmed by DNA-sequencing analysis. To generate the plasmid for the coexpression of the H63F-mutated tyrosinase and caddie, the region containing the T7 promoter and mutated *tyrC* gene was amplified with the forward primer 5′-GCACGCATGCGAAATTAATACGACTCAC-3′ (the underline indicates the SphI site) and the reverse primer 5′-CTATGCATGCCAAAAAACCCCTCAAGAC-3′ (the underline indicates the SphI site) by using the mutated plasmid as a template. The amplified fragment was digested with SphI and inserted into the same site of pET-*orf378* [11], a plasmid for the expression of the His_6_-tagged caddie protein. We chose a plasmid in which the direction of the tyrosinase and caddie genes is opposite.

### Preparation of the complex

Plasmid to overproduce the wild-type or Y98F-mutated complex has been already constructed [11,26]. The overproduction and purification of wild-type, Y98F-mutated, or H63F-mutated complex were performed by the method described previously [11].

### Crystallography

The crystallization of copper-free tyrosinase in complex with the caddie protein (including mutated complexes) was conducted by the method described previously [12,26]. The typical formula of reservoir solution to obtain the crystals was 25% PEG3350, 0.2 M NaNO_3_, and 0.1 M Na-HEPES (pH 6.5). Crystallization was performed by the sitting-drop vapor-diffusion method, in which a drop mixing 2 μL of the protein solution, 2 μL of the reservoir solution, and 1 μL of the reservoir solution containing microseeds was kept in a well containing 1 mL of the reservoir solution. For the soaking experiments, crystals with similar sizes (0.3–0.4 mm in one dimension) were chosen. The crystals of a complex between met2-form tyrosinase and wild-type or Y98F-mutated caddie were obtained by soaking the copper-free crystals in a reservoir solution containing 1 mM CuSO_4_ for about 80 h at 25 °C [26]. The crystals of a complex between deoxy-tyrosinase and the wild-type caddie protein were obtained by soaking the met2-form crystal anaerobically in an N_2_-purged reservoir solution containing 0.1 mM CuSO_4_, 10 mM NH_2_OH, 5 mM glucose, 1 μM glucose oxidase, and 5 μM catalase for 2 h at 25 °C. The latter three reagents were added to keep a low dioxygen concentration. During soaking, the crystal-containing well was filled with the purged solution, and cover glass was put over the well. Other crystals were obtained by soaking the met2-form crystal aerobically in a reservoir solution containing 0.1 mM CuSO_4_ and 10 mM NH_2_OH for the indicated times at 25 °C. Exceptionally, to generate dicopper center in the H63F-mutated complex, the copper-free crystals were directly soaked in a reservoir solution containing 1 mM CuSO_4_ and 10 mM NH_2_OH for 24 h at 25 °C.

The diffraction intensities of the crystals were collected using synchrotron radiation from the stations BL26B2, BL38B1, or BL41XU at SPring-8, Japan. Some of the crystallographic studies at SPring-8 were performed with the approval of the institution (2013A1078). When using the high levels of X-ray exposure at BL41XU, the occupancies of Cu^A-1^ and Cu^B-1^ were calculated to be higher than the values obtained at BL26B2 and BL38B1, indicating the occurrence of copper reduction by hydrated electrons. Therefore, the crystal structures obtained from BL41XU were excluded from the discussion. The crystals were frozen by liquid nitrogen or by nitrogen gas stream and mounted on the goniometer. Diffraction of each crystal was measured using a CCD camera, and the intensities were integrated and scaled using HKL2000 [53] or using the combination of Mosflm and Scala programs in the CCP4 program suite [54]. The existence of the copper ion was confirmed in anomalous difference Fourier maps. The model was refined using conventional restrained refinement methods with the CNS program [55]. A subset of 5% of the reflections was used to monitor the free *R* factor (*R*_free_) [56]. Each refinement cycle included the refinement of positional parameters, individual isotropic *B*-factors, correction using the flat bulk solvent model, and addition of solvent molecules. After the CNS refinement was converged, the model was further refined by SHELXL-97 [43]. At this stage, anisotropic temperature factors were introduced for all atoms. The distance between the copper ion and its ligand atom was not restrained. At first, occupancies of the copper ions, bridging molecules (peroxide and water), and oxygen atom bound to the caddie Tyr^98^ were refined independently. With respect to the wild-type or the Y98F-mutated complex, several restraints were imposed on the occupancies. The model was revised using the electron density map visualized by the program Xfit in the XtalView software package [57]. Occupancies of Cu^A-1^, Cu^A-2^, Cu^A-3^, peroxide, and Oζ2 atom added to Tyr^98^ at the final stage are shown in Table 2. The data collection and refinement statistics are shown in Table 1. The statistics for ST6 are worse than the values for the other five models, probably because of the low ratio of the number of reflections to that of the refined parameters. Other cryo-trapped structures obtained by the soaking at 4 °C are described in the Supporting Information.

## Supporting information

S1 Text Additional results and discussion. (DOC)

S1 Fig Information on distances between the atoms in the possible structures found in crystals. (A) is the deoxy form found in the crystal structure obtained by the anaerobic soaking for 2 h (ST1). (B) and (C) are possible structures (two kinds of μ-η^2^:η^2^-type oxy form) found in the crystal structure obtained by the aerobic soaking for 10 min (ST3). (D) to (F) are possible structures (μ-η^1^:η^2^-type oxy form, dopasemiquinone-bound half-met form, and dopaquinone-bound deoxy2 form, respectively) found in the crystal structure obtained by the aerobic soaking for 2 h (ST5). The units of distances are Å.
(TIF)

S1 Table Equivalent *B*-factors. (XLSX)

S2 Table Data collection and refinement statistics of the crystal structures obtained at 4 °C. (XLSX)

S3 Table Refined occupancies in the crystal structures obtained at 4 °C. (XLSX)

## Abbreviations

PDB: Protein Data Bank
Rms: root-mean-square
TRP1: tyrosinase-related protein 1
UV-vis: ultraviolet-visible
