# Supplementary material for: Catalytic mechanism of the tyrosinase reaction toward the Tyr98 residue in the caddie protein
Source: PLoS Biol. 2018 Dec 31;16(12):e3000077. doi: 10.1371/journal.pbio.3000077 (PMC6312201; doi:10.1371/journal.pbio.3000077)
Supplement: S1 Text — (DOC) [file pbio.3000077.s001.doc]

**Additional results and discussion**

To evaluate the effect of temperature for the progress of the tyrosinase reaction toward the caddie Tyr^98^ residue, we also determined the crystal structures, each of which was cryo-trapped after the aerobic soaking of the crystal of met2-tyrosinase complexed with the caddie protein in a buffer containing CuSO_4_ and NH_2_OH for a given time at 4°C (**ST7** to **ST10** in **S2 Table**). The crystal structures were refined using the restraints on the occupancies, as with the crystal structures obtained at 25°C. Refined occupancies are shown in **S3 Table**.

When the soaking experiments were performed at 4°C (**ST7** to **ST10**), the hydroxylation reaction proceeded more slowly than that at 25°C (**Table 2**). It should be noted that the difference between the occupancies of the Oζ2 atom and Cu^A-3^ at the late stage at 4°C (**ST8** to **ST10**) tends to be smaller than that at 25°C (**ST5**).

As shown in the previous study [1], the aggregation of the caddie protein was stimulated under the high temperature conditions, as well as the acidic pH conditions discussed in the main text. We propose the intermediates **A** to **I** in the catalytic mechanism of tyrosinase toward the caddie Tyr^98^ residue (**Fig 6**). Although intermediates **A** to **H** may coexist after the addition of Cu(II) and NH_2_OH, thermal vibration likely stimulates the conversions from **B** to **C** and from **G** to **H**, which require the long-distance movement of Cu^A^. In fact, the upshift of temperature reduced the ratio of **B** in the solution state [1], and increased the ratio of **H** in the crystalline state. However, it is unknown that whether the **H** state is a true intermediate to reproduce the deoxy form (**I**). Based on the structural information (**S1E Fig**), a proton attached to Wat^5^ would be transferred to O^distal^-derived hydroxide ion at the Wat^3^ site through the Oζ2 atom added to the Tyr^98^ residue, since distances between Wat^5^ and the Oζ2 atom and between the Oζ2 atom and Wat^3^ are short (3.6 and 2.8 Å, respectively). If so, DOPA radical complexed with the half-met form may be a true intermediate between **G** and **I** states, while **H** is a dead-end state. As discussed in the main text, the **H** state may be important for the catalytic cycle of the tyrosinase reaction.

**References**

1. Matoba Y, Kihara S, Muraki Y, Bando N, Yoshitsu H, Sakaguchi M, Kayama K, Tai H, Hirota S, Ogura T, Sugiyama M. Activation mechanism of the *Streptomyces* tyrosinase assisted by the caddie protein. Biochemistry. 2017;56: 5593–5603.
