# Supplementary figures and images for: Catalytic mechanism of the tyrosinase reaction toward the Tyr98 residue in the caddie protein
Source: PLoS Biol. 2018 Dec 31;16(12):e3000077. doi: 10.1371/journal.pbio.3000077 (PMC6312201; doi:10.1371/journal.pbio.3000077)

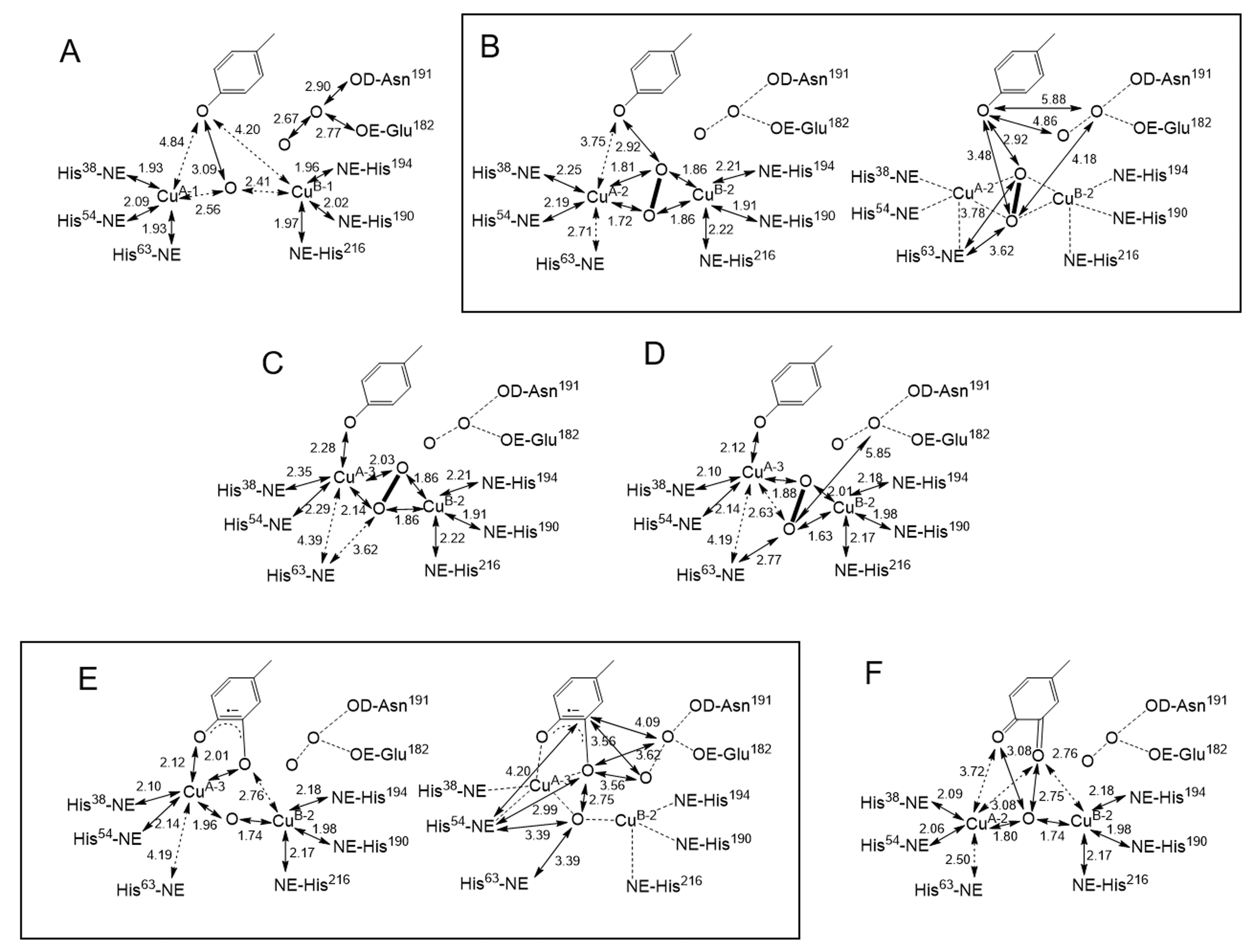

Supplement: S1 Fig — (A) is the deoxy form found in the crystal structure obtained by the anaerobic soaking for 2 h (ST1). (B) and (C) are possible structures (two kinds of μ-η2:η2-type oxy form) found in the crystal structure obtained by the aerobic soaking for 10 min (ST3). (D) to (F) are possible structures (μ-η1:η2-type oxy form, dopasemiquinone-bound half-met form, and dopaquinone-bound deoxy2 form, respectively) found in the crystal structure obtained by the aerobic soaking for 2 h (ST5). The units of distances are Å. (TIF) [file pbio.3000077.s002.tif]
